# Supplementary figures and images for: Agonal Factors Distort Gene-Expression Patterns in Human Postmortem Brains
Source: Front Neurosci. 2021 Mar 25;15:614142. doi: 10.3389/fnins.2021.614142 (PMC8027124; doi:10.3389/fnins.2021.614142)

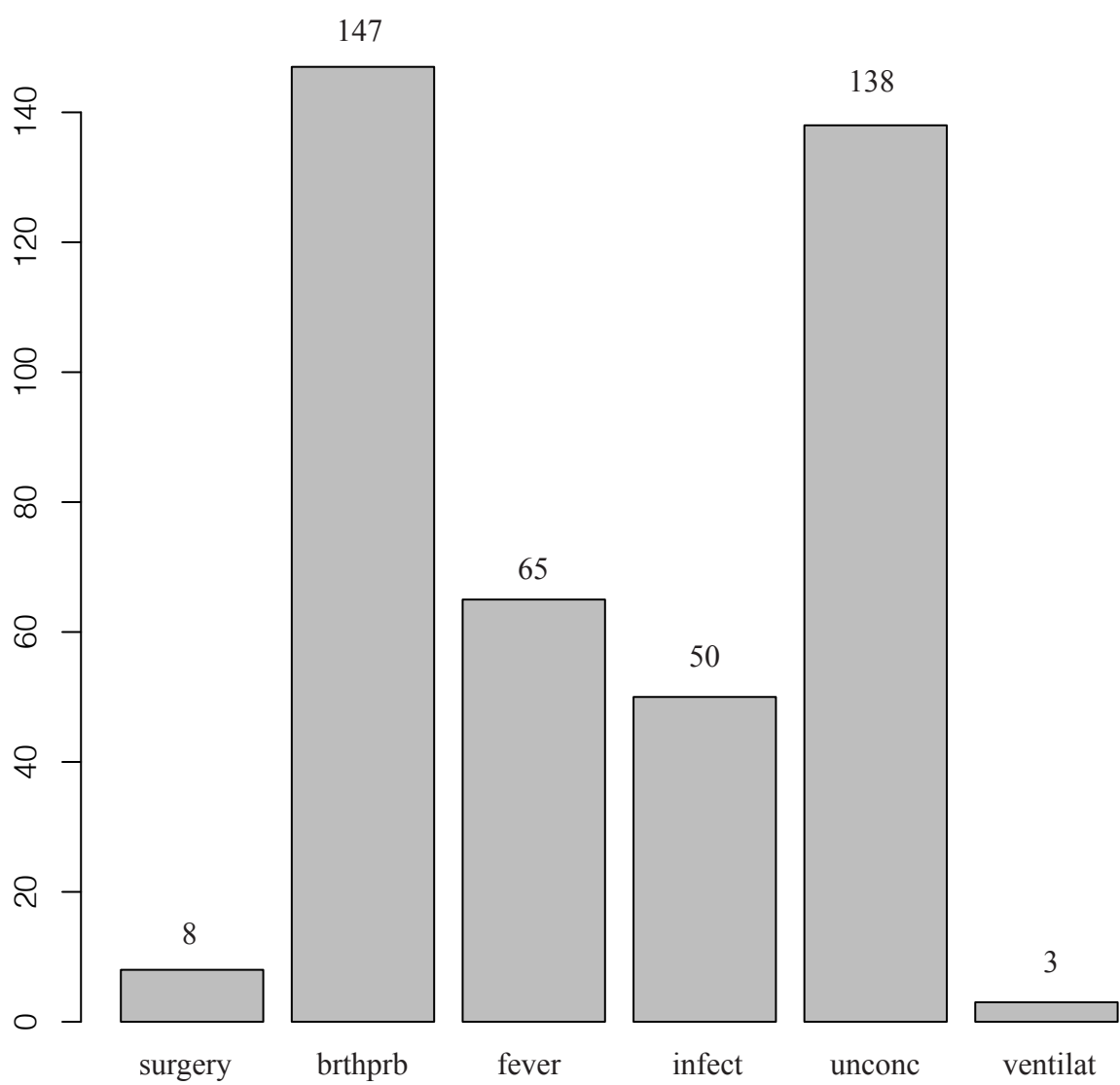

Extended figure 1. Sample size per terminal state.

Supplement: Supplementary file 4 [file Image_1.pdf]

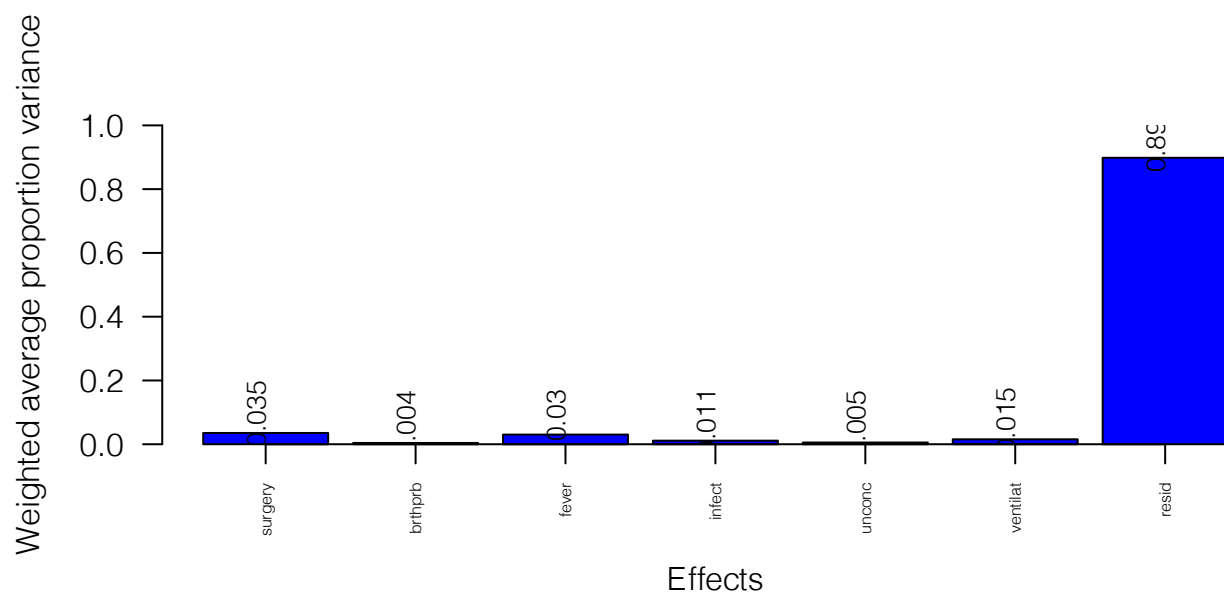

Extended Figure 2. PVCA plot of terminal states.

Supplement: Supplementary file 5 [file Image_2.pdf]
